# Heavy metal background levels and pollution temporal trend assessment within the marine sediments facing a brownfield area (Gulf of Pozzuoli, Southern Italy)

**DOI:** 10.1007/s10661-022-10480-3

**Published:** 2022-09-21

**Authors:** Giovanna Armiento, Mattia Barsanti, Raffaela Caprioli, Salvatore Chiavarini, Fabio Conte, Cinzia Crovato, Maurizio De Cassan, Ivana Delbono, Maria R. Montereali, Elisa Nardi, Luisa Parrella, Massimo Pezza, Marco Proposito, Juri Rimauro, Antonio Schirone, Fabio Spaziani

**Affiliations:** 1 ENEA Casaccia Research Centre, Department for Sustainability, Via Anguillarese 301, 00123 Roma, Italy; 2 ENEA Santa Teresa Research Centre, Località Pozzuolo di Lerici, Department for Sustainability, Lerici 19032 (SP), Italy; 3 ENEA Portici Research Centre, Department for Sustainability, Piazzale Enrico Fermi 1, (NA) 80055 Portici, Italy; 4 ISPRA, Italian Institute for Environmental Protection and Research, Via Vitaliano Brancati 48, 00144 Roma, Italy

**Keywords:** Background level, Sediment cores, Geochronology, Metals, PAHs, Brownfield

## Abstract

In this study, site-specific natural background levels (NBLs) were determined for 18 elements (Al, As, Be, Cd, Co, Cu, Cr, Fe, Hg, K, Mn, Mo, Ni, Pb, Tl, U, V, and Zn) in two sediment cores collected offshore the Bagnoli-Coroglio brownfield site (Gulf of Pozzuoli, southern Italy) to accurately assess the degree of contamination and the historical trends in Heavy Metals (HMs) enrichment. This objective was pursued taking in account the high temporal and spatial variability of the geochemical properties of the area due to the local geothermal activity. Moreover, the temporal variation of Polycyclic Aromatic Hydrocarbons (PAHs) was investigated.

^226^Ra was used as an extraordinary marker to confirm ^210^Pb dating. It especially allowed defining the geochronological framework of the sediment core closer the brownfield up to around 1500, providing compelling support to correlate the investigated elements’ occurrences with natural geogenic dynamic. Sediment samples were accurately dated and analyzed for chemical and particle size composition. The contamination factor (Cf) and the pollution load index (PLI) showed very high enrichment of Cd, Cu, Hg, Pb, and Zn. The contamination profiles of HMs and PAHs follow the same pattern in both sediment cores, increasing from deep to upper layers. The highest contamination levels for HMs and PAHs were observed between 10 and 30 cm, corresponding to the periods of most intense industrial activity. Decreasing trends of pollutants were observed in the surface layers (0–10 cm), probably affected by a natural attenuation process due to the cessation of industrial activities.

**Supplementary Information:**

The online version contains supplementary material available at 10.1007/s10661-022-10480-3.

## Introduction

The determination of heavy metal natural background levels (NBLs) in sediments is the key factor for discriminating between anthropogenic and geogenic inputs, to achieve a reliable assessment of the degree of pollution and to define appropriate environmental restoration programs for a maritime contaminated area (Matschullat et al., 2000; Rodríguez et al., 2006; Armiento et al., 2013). Moreover, in sites affected by geochemical anomalies, such as in geothermal areas, the natural concentrations of heavy metals (HMs) may have wide temporal and spatial variabilities which may make it difficult to evaluate anthropogenic inputs. Therefore, especially in such cases, it is important to achieve an accurate determination of local background values before assessing the enrichments due to human activity (Romano et al., 2015), and an approach based on the integration between a geochemical and a statistical method is recommended (Matschullat et al., 2000; Hernández-Crespo & Martín, 2015).

The Gulf of Pozzuoli, located in the Campania Region (southern Italy) between the Vesuvius volcano and the Phlegraean Fields, is an area having great environmental and economic relevance, but it is difficult to manage due to a complex contamination history. It is recognized as a Site of National Interest (SIN) by the Italian government because of the severe pollution caused by almost a century (1910–1990) of industrial activity in the Bagnoli-Coroglio brownfield area (which included the production of steel, asbestos, cement, and fertilizers). Several construction works have considerably modified the coastline over the years, mainly related to the industrial exploitation of the area. In particular, in 1920 two piers were built for the docking of vessels, and in 1962–1964, the sea section between the piers was filled using the polluted soil of the production site. Other relevant modifications were the artificial connection between the island of Nisida and the mainland, completed in 1936, and the enlargement of some littoral sections for tourism purposes (Bergamin et al., 2003). As a result, the original water circulation and sedimentation pattern were altered, and the new setting also affected the distribution of contaminants in the environment.

The investigation of HMs and organic pollutants in marine sediment cores is of great interest for assessing the temporal trend of pollution and managing the environmental risk. This is particularly relevant in coastal areas located near dismissed industrial sites (brownfields), as understanding the degree of pollution is crucial for planning potential environmental restoration.

In the last two decades, the Bagnoli-Coroglio brownfield has been the subject of several studies aimed at assessing the impact on marine sediments and planning a coherent environmental recovery program: chemical characterization of marine surface sediments and sediment cores has shown critical contamination by HMs, Polycyclic Aromatic Hydrocarbons (PAHs), and Poly Chlorinated Biphenyls (PCBs) in the proximity of the dismissed industrial site, as well as extensive dispersion of contaminants over a wider area of the Gulf of Pozzuoli (Albanese et al., 2010; Trifuoggi et al., 2017; De Vivo & Lima, 2018; Romano et al., 2018; Armiento et al., 2020).

Although the high abundances of most metals (e.g., Cd, Cu, Cr, Hg, Ni, Pb, Zn) in the sediments of the Gulf of Pozzuoli is associated with the past anthropogenic activities the contribution of the local submarine volcanic activity must also be considered. Indeed, it can affect the occurrence of potentially toxic elements (such as arsenic) deriving from deep-rising hydrothermal fluids (Signorelli, 1997; Angelone et al., 2009; Breuer & Pichler, 2013; Cinti et al., 2015), in this case related to the intense volcanic activity of the Phlegraean Fields (Aiuppa et al., 2003). Due to the local hydrostratigraphy and structural setting, the concentration of these elements may differ significantly from the average concentration in the upper surface crust. Consequently, from an environmental perspective, the trace element concentrations found in the Gulf of Pozzuoli represent a confluence of anthropogenic and geogenic inputs.

Even if numerous studies have documented the potentially adverse effects of the abovementioned elements, the time trends and discrimination between anthropogenic and geogenic inputs in sediments remain poorly documented due to the lack of both geochronological surveys and site-specific NBLs estimation.

In the present study, two sediment cores, collected in the Gulf of Pozzuoli, were characterized by grain-size measurement, and geochronological and chemical analyses. The purpose of the investigation was to provide information useful for the activities aiming at the recovery and urban re-development of the dismissed industrial site. The data obtained were processed in order to (i) estimate site-specific NBLs of 18 elements (Al, As, Be, Cd, Co, Cu, Cr, Fe, Hg, K, Mn, Mo, Ni, Pb, Tl, U, V, and Zn) through an integrated geochemical and statistical approach; (ii) achieve an assessment of the degree of HMs contamination by the contamination factor (Cf) and the Pollution load index (PLI); and (iii) describe and compare the temporal trend assessment of HMs and PAHs of the two collected sediment cores.

## Materials and methods

### Sediment sampling

**Fig. 1:**
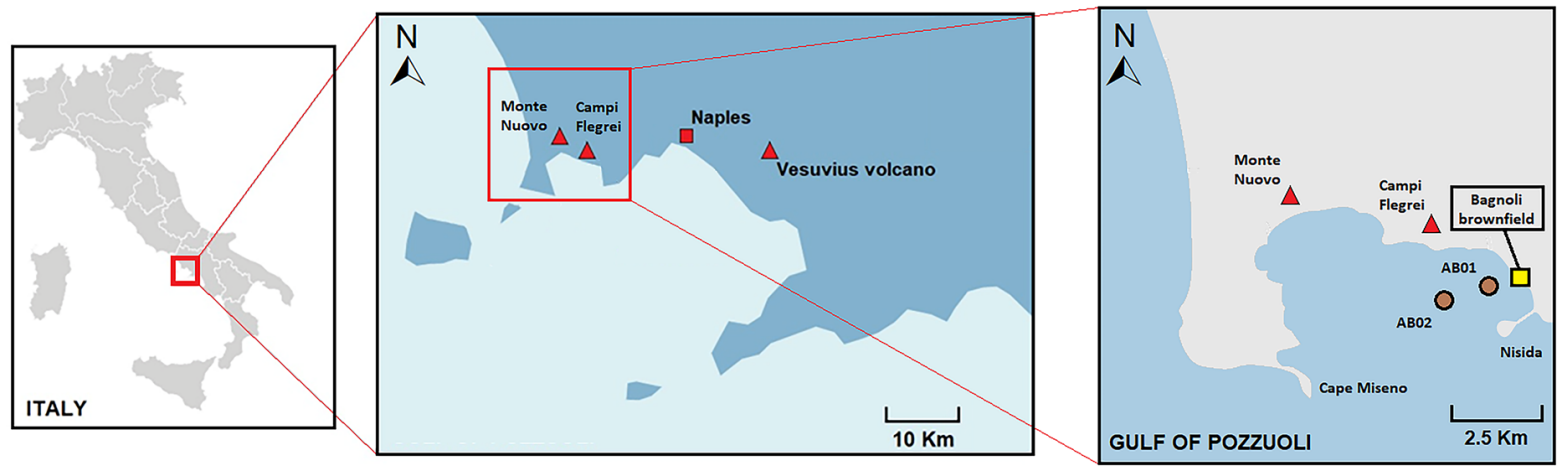
Location of the two sediment cores sampled (AB01 and AB02) in the Gulf of Pozzuoli (Campania, southern Italy)

Two sediment cores (AB01 and AB02) were collected in December 2018 in front of the Bagnoli-Coroglio brownfield site and in a central area of the Gulf of Pozzuoli, at 55- and 65-m depth, respectively (Fig. 1). Undisturbed sediment cores, with their aqueous component at the water-sediment interface, were collected using a Carma^®^ Corer SW-104. The location and characteristics of the sediment cores are reported in Table 1.

**Table 1:** Location, sea depth, and length of the sediment cores

**Core**	**Latitude**	**Longitude**	**Seabed depth at the sampling point (m)**	**Length of the sediment core (cm)**
AB01	40°48.150′ N	14°08.913′ E	55	109
AB02	40°48.198′ N	14°07.157′ E	65	68

Both sediment cores were cut at 1-cm intervals, and each slice was further subdivided into subsamples for different determinations; particle size analysis, chemical analyses (organic compounds and metals), and radiometric measurements were performed on 39 samples from core AB01 core and 34 samples from core AB02.

### Radiometric measurements and geochronological determination

Radiometric analyses were performed by gamma spectrometry, using two ORTEC AMETEK Gamma-X HPGe coaxial radiation detectors. Calibration and measurement procedures have been described in Delbono et al. (2016). The quality check performances for both detectors are presented in Table ESM 1 (Electronic Supplementary Material). The radionuclides considered for dating are ^210^Pb and ^226^Ra. The excess ^210^Pb (^210^Pb_ex_) activity is calculated as the difference between the total ^210^Pb and the fraction in equilibrium with the parent radionuclide ^226^Ra. Calculations for the chronology of the sediment layers were performed using the mass depth (g cm^−2^) to account for the compaction of the sediment layers (Cutshall et al., 1983).

Of all the daughter products of the ^238^U, ^235^U, and ^232^Th series radionuclides, ^210^Pb is the most used isotope for dating samples (sediments, ice cores, corals, etc.) ranging from one year to ~110 years old (Sanchez-Cabeza and Ruiz-Fernàndez, 2012 and references therein). To determine the age of each layer of the two sediment cores (AB01 and AB02), the Constant Rate of Supply (CRS) model was applied (Appleby, 2001). The assumptions for obtaining reliable ^210^Pb-based ages require validation with another independent parameter (Smith, 2001): to attain this aim, we apply ^226^Ra activities in the present work. In fact, sediments ejected from volcanic activities at Vesuvius are marked by a high ^226^Ra concentration (Voltaggio et al., 2004), and its recent historical activity (1631–1944) is well known and continuous (Scandone et al., 2008). Another volcanic activity in the area, relevant for dating, was the uplift of Monte Nuovo in 1538 at Pozzuoli (Guidoboni & Ciuccarelli, 2011). Thus, in this volcanic environment, the ^226^Ra activity has proven to be an extraordinary temporal marker.

### Particle size analysis

Sediment core sub-samples were washed with deionized water and treated with hexametaphosphate for 24 h to disaggregate the phyllosilicate agglomerates, typical of the clay fraction. Particle size analyses were carried out with a Sympatec Helos/KF laser diffractometer: this instrument returns the volume percentages of the different particle size fractions present in the sediment sample. In this study, particle size data are reported as three main fractions: sand (> 63 µm), silt (between 63 and 4 µm), and clay (< 4 µm).

### Analytical determinations

The samples were placed in 500 mL PEHD containers and stored at a temperature of 4 °C until arrival at the laboratory. For each sample, three aliquots were isolated and pre-treated according to the type of analysis: the aliquot used for the analysis of HMs (except Hg) was dried in a thermoventilated oven at a temperature below 40 °C; the aliquot intended for the analysis of organic compounds was freeze-dried; the aliquot for the analysis of Hg was air-dried in a fume hood.

The total fraction of HMs in the sediments was obtained by microwave-assisted acid digestion according to EPA 3052 method. The analytical determination of metals was carried out using the following methods and instruments: EPA 6010d method, for the determination of Al and Fe, using an ICP-OES (Perkin Elmer Optima 2000 DV); EPA 6020b method, for the determination of As, Be, Cd, Co, Cu, Cr, K, Mn, Mo, Ni, Pb, Tl, U, V, and Zn, using an ICP-MS (Agilent 7800); EPA 7473 method, for the determination of Hg, using an automatic solid/liquid mercury analyzer (FKV AMA-254). The quantitation limits, expressed as mg/kg (dry weight), were 0.05 for As, V, and Pb; 0.03 for Cd; 0.5 for Cr; 0.3 for Cu and Ni; 1.0 for Zn; 50 for Al; 5 for Fe; and 0.005 for Hg. The analytical quality control was executed using reagent blanks, replicate measurements, and standard reference materials. Specifically: a reagent blank was produced and analyzed for every set of samples digested; about 20% of samples were analyzed in triplicate, and the results confirmed that the value variations were within 5%; two certified reference materials (PACS-3 and MESS-4), were processed and analyzed using the same procedures applied to sediment samples and the recovery rates ranged from 80% to 105%.

The sixteen PAHs designated as High Priority Pollutants by EPA were extracted, after the addition of surrogate standards, according to EPA 3545a method, using an Accelerated Solvent Extractor (ASE 200 Dionex); then, the silica gel cleaning technique was used as described in EPA 3630 method. The analytical determination of PAHs was performed, according to the EPA 8270D method, with an Agilent 7890A-5975C GC-MS system equipped with a DB 5MS capillary column (30 m × 0.25 mm × 0.25 µm film thickness). The quantitation limit for all compounds was 0.1 µg/kg. The analysis was performed in SIM mode and using the internal standard method to plot the calibration curve. The analytical quality assurance of PAHs measurements was carried out using the following certified reference materials: CRM 535, SRM 1944, and SRM 1941b.

All the quantitation limits mentioned above were calculated as dry weight.

### Data analysis

The dataset was processed by principal component analysis (PCA) in order to further understand the correlation between parameters (HMs and/or PAHs) and to distinguish samples that have similar distribution patterns. Prior to PCA processing, data were standardized to remove differences in scale between the variables. To standardize the variables, the mean was subtracted from each observed value and the result was divided by the standard deviation. All the statistics were elaborated using Past (version 4.06).

### Natural background levels determination

A combined approach, including geochemical and statistical method, based on three steps was applied to determine the site-specific NBLs for 18 metals (Al, As, Be, Cd, Co, Cr, Cu, Fe, Hg, K, Mn, Mo, Ni, Pb, Tl, U, V, Zn). In the first step, geochronological information was used to distinguish between human-influenced (i.e., post-industrial time) and uncontaminated (i.e., pre-industrial time) layers in both sediment cores. In the second step, the two original chemical datasets from the sediment cores were reduced and gathered in a single “anthropogenically undisturbed” dataset: to achieve this purpose, several statistical tests were applied to normalize collected data, using ProUCL5.1 (US EPA), and boxplots were created to graphically identify and remove statistical outliers. Moreover, values related to possible high natural geochemical anomalies, such as samples attributed to the time of Monte Nuovo eruption (1538 A.D.), characterized by a peculiar pattern of element concentrations, were removed: while the almost continuous Vesuvius activity between 1631 and 1944 A.D. may be considered a component of NBLs, this eruption should be considered a unique event. Finally, in the third step, the background concentrations were assessed as upper threshold values of the natural variability of each element and calculated from the mean (µ) and standard deviation (σ) of the dataset obtained as µ + 2σ (Matschullat et al., 2000).

### Assessment of the degree of contamination

In order to assess the degree of contamination in the collected sediment cores, two widely used indices were calculated: the contamination factor (Cf) (Hakanson, 1980), and the pollution load index (PLI) (Tomlinson et al., 1980).

The Cf of each element in a sample (layer) from the sediment cores was determined as the ratio of the element concentration (*C*_n_) obtained from the chemical analysis to the site-specific baseline/background value for the same element (*B*_n_) (i.e., in the present study, the NBL value resulting from the abovementioned data reduction/processing):

$$Cf=\frac{{C}_{n}}{{B}_{n}}$$

The Cf values are classified according to four categories: Cf < 1, low contamination; 1 ≤ Cf < 3, moderate contamination; 3 ≤ Cf < 6, considerable contamination; Cf ≥ 6, very high contamination.

Finally, the multi-element index PLI was used to evaluate the overall sediment contamination, as a result of the contribution of all “n” metals investigated:

$$PLI=\sqrt[n]{{Cf}_{1}\times {Cf}_{2}\times ...{Cf}_{n}}$$

According to its peculiar classification, PLI > 1 indicates a contaminated sample, whereas PLI < 1 indicates an uncontaminated sample.

## Results and discussion

### Geochronological analysis

**Fig. 2:**
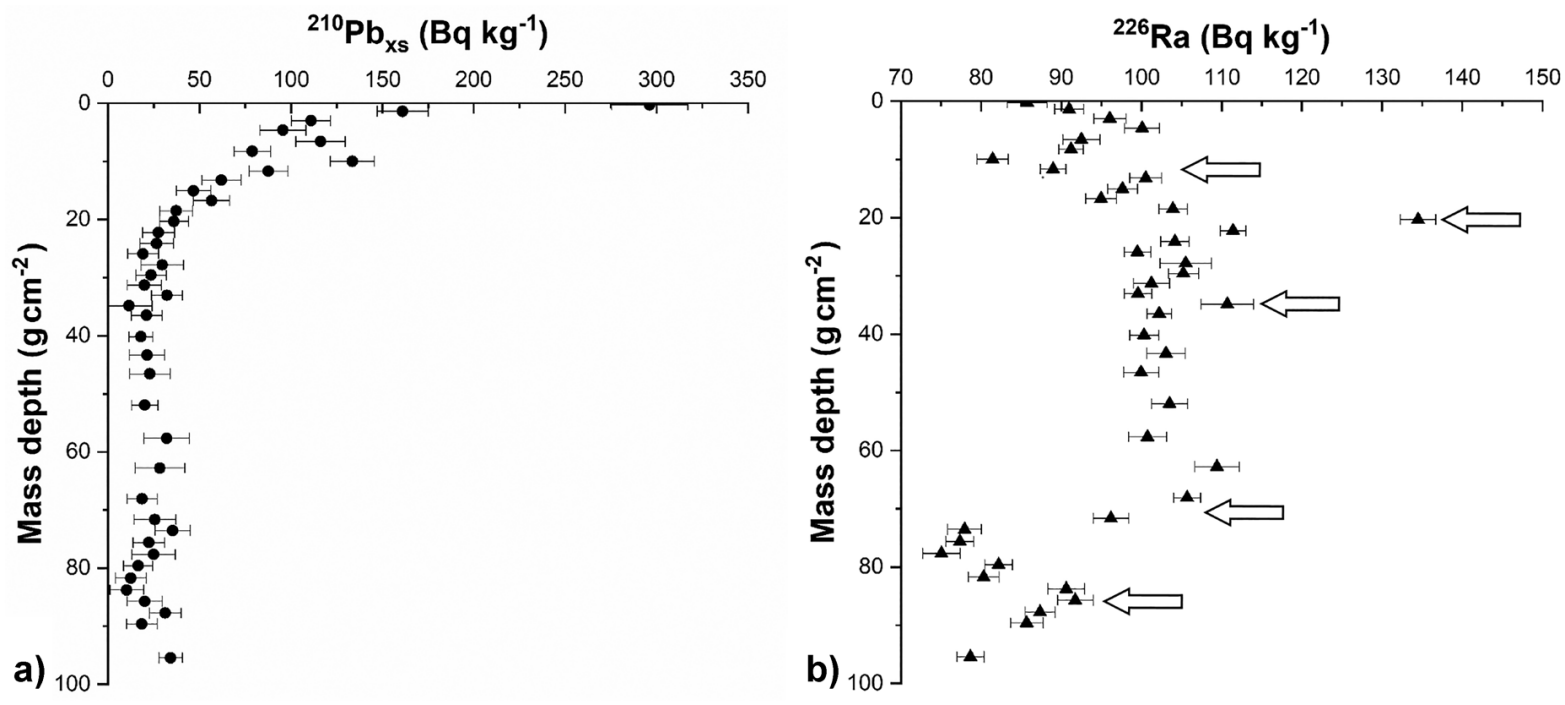
Activity of ^210^Pb_ex_ [**a**] and ^226^Ra [**b**] along the AB01 sediment core. The arrows indicate, from top to bottom, the eruptions of 1944, 1906, and 1822, the beginning of eruptive activity at Vesuvius in 1631, and the formation of Monte Nuovo at Pozzuoli in 1538

Figure 2 shows the radiometric activity profiles of ^210^Pb_ex_ and ^226^Ra (Bq kg^−1^) along the AB01 sediment core.

The ^210^Pb_ex_ profile (Fig. 2a) does not show a regular exponential trend in the superficial layers (0–20 g cm^−2^). Below, ^210^Pb_ex_ remains almost constant, without ever reaching zero (23 ± 7 Bq kg^−1^, mean value and standard deviation calculated below 22 g cm^−2^). This peculiarity can be explained by the presence, in the Puteolan area, of gaseous radon (^222^Rn) released from the underlying rock layers, which generates concentrations of about 20–60 kBq m^−3^ (Sabbarese et al., 2020). This input of ^222^Rn, decaying into ^210^Pb, adds a constant value of about 23 ± 9 Bq kg^−1^ to the fraction of ^210^Pb in equilibrium with ^226^Ra in the deep part of the AB01 sediment core. The study of the ^226^Ra activity profile corroborates the ^210^Pb dating method in the upper layers of the sediment core and provides very precise information on the progress of sedimentation over the last few centuries, greatly increasing the dating range available with the ^210^Pb method (usually ~110 years). The key factor that allows us to obtain this information is the ^226^Ra content present in Vesuvius emissions during eruptions (Voltaggio et al., 2004). Figure 2b shows, in particular, the clear peaks of ^226^Ra activity related to the three main eruptions of Vesuvius in the period 1944–1800 (1944, 1906, and 1822) and the phase related to the beginning of volcanic activities that have continued since 1631 (Scandone et al., 2008). Near the bottom of the sediment core (~85 g cm^−2^), a different radiometric signature indicates volcanic activities related to the formation of Monte Nuovo in 1538 (Guidoboni & Ciuccarelli, 2011).

The particular trend of ^226^Ra along the AB01 sediment core allowed us to accurately determine the age of all the sediment layers (Table 2). Indeed, the five temporal markers shown in Fig. 2 allow evaluating the mass accumulation rate ω [g cm^−2^ a^−1^] for each time interval, over a much longer period than allowed by the ^210^Pb method. By evaluating ω over the 5 intervals (2018–1944, 1944–1906, 1906–1820, 1820–1631, 1631–1538), it is possible to calculate the mean value of mass accumulation rate ω and its standard deviation: *ω* = 0.18 ± 0.01 g cm^−2^ a^−1^. Given the stability of the mass accumulation rate downcore, a very reliable dating can be determined for the entire sediment core, assuming a constant sediment accumulation rate.

**Table 2:** AB01 sediment core chronology

**Layer**	**Mass depth**	**Year**	**u(Year)**	**Layer**	**Mass depth**	**Year**	**u(Year)**	**Layer**	**Mass depth**	**Year**	**u(Year)**
*cm*	*g cm*^−*2*^	*a*	*a*	*cm*	*g cm*^−*2*^	*a*	*a*	*cm*	*g cm*^−*2*^	*a*	*a*
0–1	0.2	2016	1	29–30	25.0	1880	4	58–59	49.9	1741	8
1–2	0.8	2013	1	30–31	25.9	1875	4	59–60	50.7	1736	8
2–3	1.4	2010	1	31–32	26.8	1870	4	60–62	52.0	1729	8
3–4	2.2	2007	1	32–33	27.8	1865	4	62–64	53.9	1719	7
4–5	3.0	2005	1	33–34	28.7	1860	4	64–66	55.9	1708	6
5–6	3.8	2003	1	34–35	29.6	1856	4	66–68	57.7	1698	6
6–7	4.6	2001	1	35–36	30.4	1851	4	68–70	59.4	1689	6
7–8	5.6	1997	2	36–37	31.3	1846	4	70–72	61.0	1679	5
8–9	6.6	1993	2	37–38	32.1	1842	3	72–74	62.8	1670	5
9–10	7.4	1990	2	38–39	33.0	1837	3	74–76	64.6	1660	4
10–11	8.3	1987	2	39–40	34.0	1832	3	76–78	66.3	1651	4
11–12	9.1	1982	2	40–41	34.8	1827	3	78–80	68.1	1641	4
12–13	10.0	1975	3	41–42	35.7	1822	3	80–82	69.9	1631	3
13–14	10.8	1967	4	42–43	36.5	1818	3	82–84	71.6	1621	4
14–15	11.7	1960	4	43–44	37.2	1814	3	84–86	73.5	1611	4
15–16	12.5	1954	5	44–45	38.2	1808	3	86–88	75.6	1600	5
16–17	13.2	1944	3	45–46	39.2	1803	3	88–90	77.7	1588	5
17–18	14.1	1939	3	46–47	40.2	1798	4	90–92	79.6	1583	5
18–19	15.1	1934	3	47–48	41.0	1793	4	92–94	81.7	1571	5
19–20	15.9	1929	3	48–49	41.9	1788	4	94–96	83.8	1560	4
20–21	16.7	1925	3	49–50	42.7	1784	4	96–98	85.7	1549	4
21–22	17.6	1921	3	50–51	43.3	1780	4	98–100	87.7	1538	3
22–23	18.5	1916	3	51–52	44.0	1777	4	100–102	89.6	1528	4
23–24	19.5	1911	3	52–53	44.8	1772	5	102–104	91.5	1518	4
24–25	20.3	1906	3	53–54	45.7	1767	5	104–106	93.4	1507	5
25–26	21.3	1901	3	54–55	46.6	1762	5	106–108	95.4	1496	5
26–27	22.2	1895	3	55–56	47.4	1758	5				
27–28	23.2	1890	3	56–57	48.2	1750	8				
28–29	24.1	1885	3	57–58	49.0	1745	8				

*u(Year*) age uncertainty

**Fig. 3:**
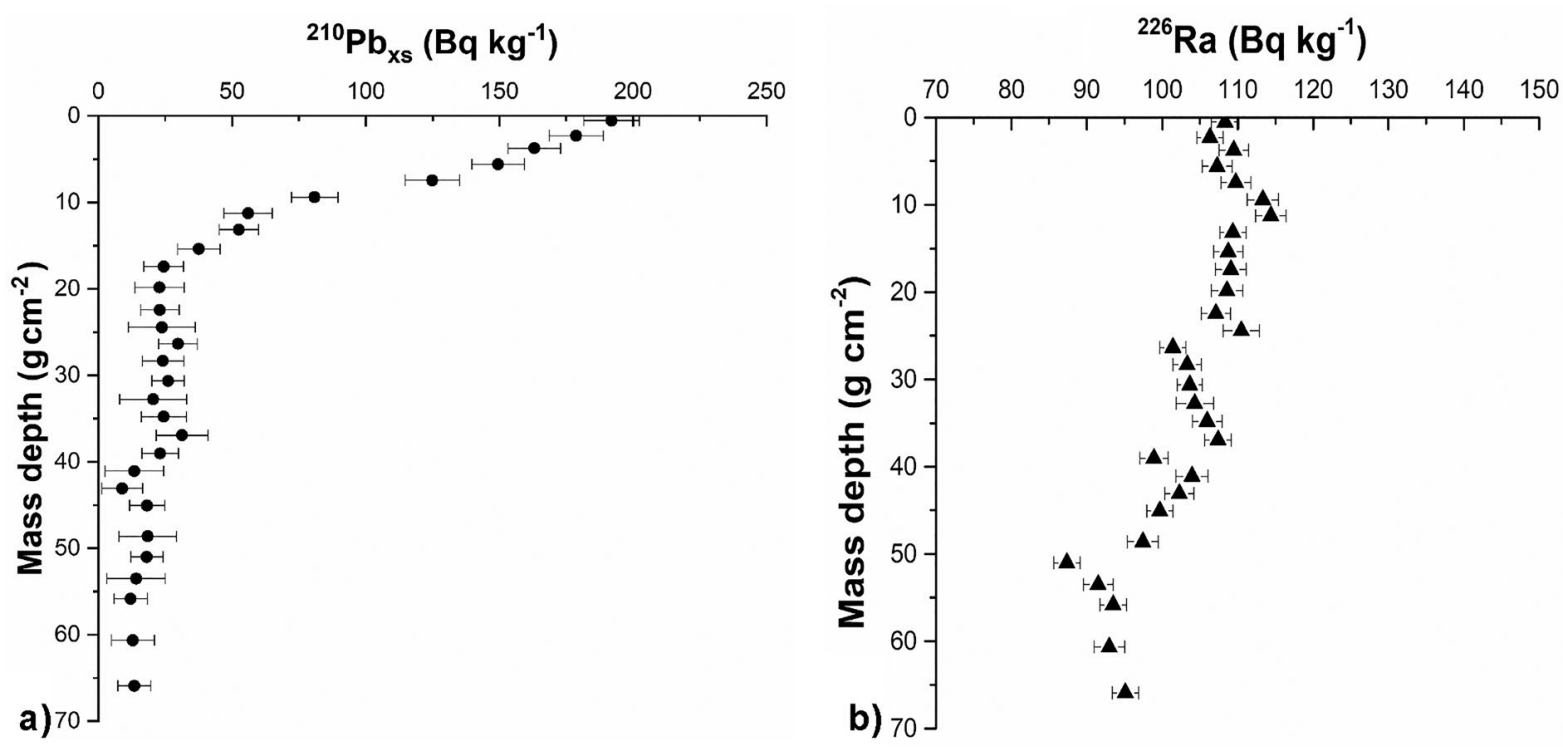
Activity of ^210^Pb_ex_ [**a**] and ^226^Ra [**b**] along the AB02 sediment core

Figure 3 shows the activity profiles of ^210^Pb_ex_ and ^226^Ra (Bq kg^−1^) along the AB02 sediment core. In this core, the activity profile of ^210^Pb (Fig. 3a) is almost regular, and the contribution of ^222^Rn from the deep crustal layers to the surface can be estimated below 20 g cm^−2^ as 20 ± 6 Bq kg^−1^ similarly to the AB01 sediment core. Although AB02 is located quite close to AB01, and it is reasonable that the contributions of ^226^Ra from Vesuvius are similar, this sediment core (Fig. 3b) does not show any peak of ^226^Ra as sharply as the AB01 sediment core, to be used as a time marker.

Thus, no signals were found in the AB02 sediment core to confirm the dating provided by ^210^Pb using the CRS model. Furthermore, both the shape of ^210^Pb_ex_ profile and the absence of peaks in ^226^Ra values suggest that post-depositional processes played an important role in this core. The presence of a stationary, partially mixed layer in the first 6 g cm^−2^ near the surface may explain these trends. The main consequence is that, in this sediment core, it is not possible to associate one-year sediment deposition with a single well-defined depth: sedimentary contributions are distributed over a depth range of several layers. However, radionuclide analysis allows us to conclude that below a depth of 20.7 g cm^−2^ (21 cm) interactions with the surface during the past 110 years (5 half-lives of ^210^Pb) are negligible.

### Particle size analysis

**Fig. 4:**
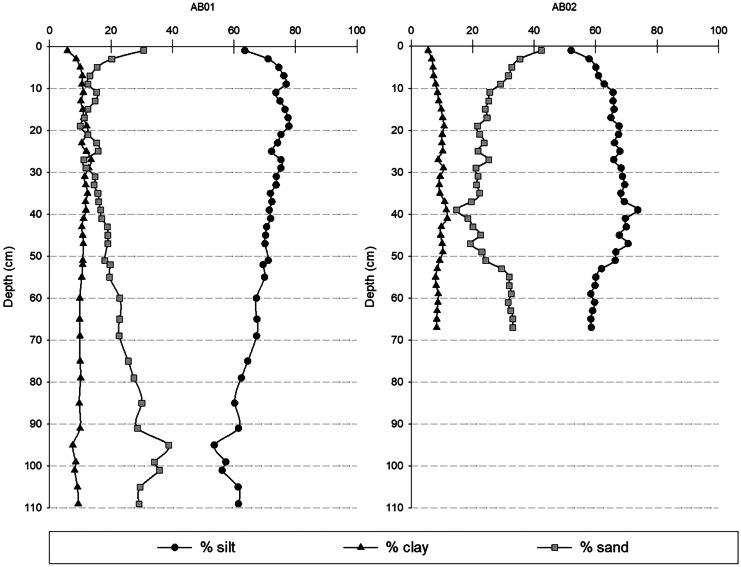
Volume percentages of sand, silt and clay along sediment cores AB01 and AB02

The results of the particle size analysis are summarized in Fig. 4. The AB01 sediment core is characterized by medium to coarse silt sediments, according to the classification of Wentworth (1922), and a volume percentage of silt corresponding to 70% ± 6%. Along the core AB01, the sand fraction is 20% ± 7%, with a gradual increase in sand from 60 cm to the bottom of the core, with a maximum of 35% sand at 100 cm depth, corresponding to the local volcanic activity in the year 1538. The clay fraction is regular throughout the core, always around 10% ± 1%. Sediment core AB02 is characterized by coarse silt sediment, with the only exception of the surface classified as very fine sand. The sediment core shows silt percentages of 65% ± 5% and the sand fraction of 26% ± 6%, with high sand values in the upper 9 cm (up to 42% at the surface) and values of 30% or more from 52 cm to the bottom of the core. The clay fraction is regular throughout the core with a volume percentage of 9% ± 1%.

The fine sediment fraction, compared to coarse particles, generally contains greater concentrations of trace metals due to its larger surface area and higher cation exchange capability (Zhang et al., 2014). However, the two sediment cores studied show almost the same reciprocal textural fraction percentages, suggesting a similar behaviour of the pollutants.

### Determination of natural background levels

The NBLs of the elements, reported in Table 3, were calculated using a data set of 30 concentration values for each metal, corresponding to layers of pre-industrial time of both sediment cores AB01 and AB02. The values obtained differ from the average shale values (a material often used as a reference matrix for calculating the Cf and other indices related to the assessment of HMs pollution), except for Al, Cd, Hg, and Mn concentrations. We also compared our NBLs with geochemical background values published by Sprovieri et al. (2020), calculated on top-core sediments from the southern Campanian shelf, showing significant differences for many elements (e.g., Al, Cd, and Pb). This evidence is somehow expected, given the completely different nature of the rocks characterizing the two areas: volcanic in the northern part (a complex volcanic area where volcanic and sedimentary processes strongly interacted during the Late Quaternary), and sedimentary in the southern part of the Campania Region. This comparison is particularly relevant because it highlights that even geochemical backgrounds calculated on different locations of the same region can vary widely, as they are closely related to the geology of underlying rocks. Consequently, the availability of a site-specific background is essential to improve the reliability of the pollution assessment as much as possible.

**Table 3:** Summary statistics of the samples used to calculate natural background levels (NBLs), and comparison with other background values from the literature

Element	Mean	Std. dev.	Skewness	Kurtosis	NBL (this study)	Southern Campania shelf*	Average shale**
	mg/kg	mg/kg			mg/kg	mg/kg	mg/kg
Al	84,149	2044	−0.14	−0.21	88,237	48,238	80,000
As	24.6	1.32	0.55	0.13	**27.2**	21.49	13
Be	8.19	0.73	−1.89	6.52	**9.66**		3
Cd	0.21	0.04	0.87	1.00	0.294	0.16	0.30
Co	7.90	0.81	0.34	−1.19	9.52		19.0
Cr	17.3	2.99	0.23	−1.28	23.3	51.05	90
Cu	18.4	4.27	−0.65	−0.35	26.9	24.57	45
Fe	26,378	2151	−0.44	0.82	30,680	36,448	47,200
Hg	0.21	0.12	0.28	−1.46	0.448	0.07	0.4
K	47,976	2693	0.23	−0.38	53,363		26,600
Mn	651	64.2	−0.31	−0.34	779		850
Mo	3.31	0.58	0.07	−1.74	4.48		2.6
Ni	10.4	1.19	−0.04	−0.74	12.8	28.91	68
Pb	64.2	10.5	0.31	−0.87	85.2	21.43	20
Tl	1.51	0.15	0.22	−1.54	**1.81**		1.4
U	7.11	0.83	0.30	−0.67	8.77		3.7
V	87.2	6.90	−0.27	−0.69	**101**	75.23	13
Zn	70.3	4.78	0.61	−0.29	79.8	80.39	95

^*^From Sprovieri et al. (2020)

^**^From Turekian and Wedepohl (1961)

The values in bold exceeding the Italian contamination threshold values for soils (residential use) set by legislation (D.Lgs. 152/06)

For some elements, NBLs exceed the limit values defined by Italian legislation (Legislative Decree 152/06) concerning the contamination threshold for soils (residential use): As (27.2 vs. 20 mg/kg), Be (9.66 vs. 2 mg/kg), Tl (1.81 vs. 1 mg/kg), V (101 vs. 90 mg/kg). These elements are known to be usually enriched in volcanic rocks and particularly in those of the Phlegrean Fields. Among them, As is the best-known element responsible for geogenic enrichments in the area, mainly associated with deep rising fluids of the active hydrothermal system (Aiuppa et al., 2003; 2006; Cicchella et al., 2005). High values of Be are commonly found in the Roman and Neapolitan volcanic provinces of central and southern Italy (Armiento et al., 2013), where felsic and alkaline bedrock mainly accounts for the high values, up to about 20 mg/kg for soils and stream sediments (Salminen et al., 2005). Volcanic rocks in the area also influence Tl concentrations, with background values similar to those proposed by Cicchella et al. (2005) for Vesuvian (2.7 mg/kg) and Phlegrean (1.7 mg/kg) areas. The concentrations of V also reflect a geogenic source, being in line with those measured for volcanic products (Tomlinson et al., 2012; Belkin et al., 2016) and for the background values of soils in the area (Cicchella et al., 2005), as well as with its geochemical pattern in soils of the Campania Region (Zuzolo et al., 2017). These elements belong to what is defined in the literature as a “geothermal suite” of contaminants (Webster & Nordstrom, 2003).

These results confirm the importance of defining site-specific background values, particularly when any contamination needs to be assessed against standard intervention limits set by the law. A comparison of sites under evaluation with NBLs may help managers make decisions about appropriate risk management, remedial actions, planning, and health protection.

### Pollution degree assessment

The descriptive statistical parameters for HMs and PAHs distribution in both sediment cores AB01 and AB02 are summarized in Tables 4 and 5, respectively.

**Table 4:** Summary statistics of HM and PAH concentrations in AB01 sediment core

AB01	Min	Max	Mean	Std. dev.	25th	Median	75th
mg/kg							
Al	70,230	87,898	81,193	4582	78,312	81,753	84,909
As	22.6	51.5	31.6	9.00	24.5	27.1	39.0
Be	5.17	11.7	8.03	1.08	7.17	8.21	8.57
Cd	0.128	2.69	0.649	0.714	0.186	0.283	0.893
Co	4.68	10.5	7.73	1.27	7.08	7.45	8.55
Cr	9.10	56.4	22.3	12.9	14.6	15.5	28.8
Cu	9.70	115	44.2	34.7	20.6	24.0	69.2
Fe	18,042	73,176	33,128	13,872	25,309	26,654	33,547
Hg	0.100	2.77	0.926	0.882	0.270	0.390	1.540
K	38,128	59,187	47,160	4564	44,304	47,947	49,458
Mn	452	1421	749	226	609	667	780
Mo	2.73	4.35	3.67	0.450	3.39	3.71	4.02
Ni	6.30	20.9	11.4	3.10	9.60	10.5	13.1
Pb	43.1	648	184	177	60.0	81.6	339
Tl	1.32	1.93	1.64	0.140	1.57	1.64	1.70
U	5.49	12.7	7.14	1.59	6.13	6.73	7.99
V	58.4	111	86.0	10.1	80.9	83.7	91.3
Zn	51.4	1224	307	359	70.6	79.9	583
Σ PAHs	91.5	301,607	51,767	87,111	436	1643	56,659
L-PAHs	44.5	26,567	4150	7358	124	203	5629
H-PAHs	0.04	275,040	47,618	79,894	266	1459	72,780

**Table 5:** Summary statistics of HM and PAH concentrations in AB02 sediment core

AB02	Min	Max	Mean	Std. dev.	25th	Median	75th
mg/kg							
Al	76,000	88,000	83,003	2551	81,500	82,700	84,275
As	22.5	30.7	25.1	1.70	24.0	25.1	25.7
Be	7.61	9.32	8.29	0.44	7.97	8.26	8.59
Cd	0.16	0.33	0.23	0.05	0.20	0.21	0.26
Co	6.93	10.2	8.92	0.82	8.46	9.03	9.51
Cr	14.0	41.4	23.8	7.50	20.0	20.6	24.8
Cu	9.50	31.6	19.8	6.40	16.3	18.8	23.2
Fe	23,486	36,520	28,853	3080	26,547	28,503	30,699
Hg	0.03	0.98	0.28	0.24	0.10	0.16	0.45
K	42,800	55,200	46,121	2758	44,400	45,250	46,350
Mn	544	876	699	69.0	647	697	742
Mo	2.37	3.91	2.81	0.27	2.66	2.74	2.92
Ni	7.80	15.4	11.9	1.80	10.9	11.9	13.1
Pb	46.7	155	79.4	34.1	56.0	65.7	91.1
Tl	1.29	1.63	1.40	0.07	1.36	1.39	1.45
U	6.00	8.74	7.34	0.66	6.89	7.45	7.78
V	76.4	98.8	92.9	4.10	91.0	93.1	95.4
Zn	64.4	220	98.4	52.4	67.7	70.3	107
Σ PAHs	64.3	47405	14,364	15,866	695	7734	26,663
L-PAHs	40.3	4399	1115	1232	108	743	2010
H-PAHs	24.0	43,006	13,249	14,662	588	6992	24,653

L-PAHs (low molecular weight PAHs): sum of naphthalene, acenaphthene, acenaphthylene, fluorene, phenantrene, and anthracene; H-PAHs (high molecular weight PAHs): sum of fluoranthene, pyrene, benzo[a]anthracene, chrysene, benzo[b]fluoranthene, benzo[k]fluoranthene, benzo[a]pyrene, indeno[1,2,3-cd]pyrene, dibenzo[a,h]anthracene, and benzo[g,h,i]perylene; ΣPAHs (total PAHs): sum of 16 PAHs

L-PAHs (low molecular weight PAHs): sum of naphthalene, acenaphthene, acenaphthylene, fluorene, phenantrene, and anthracene; H-PAHs (high molecular weight PAHs): sum of fluoranthene, pyrene, benzo[a]anthracene, chrysene, benzo[b]fluoranthene, benzo[k]fluoranthene, benzo[a]pyrene, indeno[1,2,3-cd]pyrene, dibenzo[a,h]anthracene, and benzo[g,h,i]perylene; ΣPAHs (total PAHs): sum of 16 PAHs

In AB01 core, the mean concentration of As, Cd, Cu, Fe, Hg, Pb, and Zn are higher than the NBLs, while in AB02 core only the mean concentration of Pb and Zn exceed the NBLs. Comparing the sediment cores, AB01 show concentration values higher than AB02. In AB01 observed, the maximum values are for As, Cd, Cr, Cu, Fe, Hg, Mn, Pb, and Zn. The results show concentration values of both organic and inorganic pollutants in agreement with the results of previous studies conducted on the Gulf of Pozzuoli and on the Bagnoli-Coroglio bay from 1978 to the present (Table 6).

**Table 6:** Range of metals (mg/kg) and PAHs (mg/kg) in marine sediments of Gulf of Pozzuoli and Bagnoli-Coroglio coastal area

Area	Sample	Al	As	Be	Cd	Co	Cr	Cu	Fe	Hg	Mn	Ni	Pb	V	Zn	PAHs	Reference
Gulf of Pozzuoli	Surface sediment	29,955–87,205	18.7–83.7		0.26–0.96		11.2–87.1	6.6–60	21,893–102,294	0.01–1.10		4.2–28.3	47.3–249	61.8–200	94.8–713	172.60–746008	Armiento et al. (2020)
Bagnoli-Coroglio	Sediment core	27,725–201,371	27.8–845		0.24–26.1		8–623	5.4–210	21,579–209,372	0.001–7.5		4.04–94	16.1–2486	42.03–360	71.3–8535	49.10–2839450	Armiento et al. (2020)
Bagnoli-Coroglio	Sediment core	1881–34,529	50.2–248		0.03–113		4.7–50.2	2.0–216	20,057–89,284	0.01–10.2		3.9–41.1	26–2811	52.2–204	63.1–6298	0.10–67.32	Romano et al. (2018)
Bagnoli-Coroglio	Sediment core	5401–23,647	9.5–836		0.03–0.31		2.8–46.6	2.7–271	13,230–38,919	0.01–0.55		1.5–99.9	24.1–829	64.5–132	50.6–389	0–7.99	Romano et al. (2018)
Bagnoli-Coroglio	Sediment core	10,007–46372	22.4–137		0.01–0.06		1.6–14.5	3.2–29.6	15,227–25,999	0.01–0.04		2.6–72.6	24.1–131	44.5–90.6	92.7–309	0–0.25	Romano et al. (2018)
Gulf of Pozzuoli	Surface sediment		12.3–100		0.0–0.7		0.5–49.5	3.5–86.2	10,500–66,800	0.0–25.3	20–1353	0.0–35.4	11.5–378		42.1–870		Trifuoggi et al. (2017)
Gulf of Pozzuoli	Surface sediment	700–39,000	1.4–73	0.01–14.1	0.01–44	1.3–73	1.9–142	2.9–408	6000–116,000	0.01–8.3	277–9709	3.41–280	21–3446	15.6–575	90–5185	0.05–2947	Albanese et al. (2010)
Gulf of Pozzuoli	Surface sediment		13.0–176		0.05–0.33		10.0–103	13.9–287	18,173–79,043	0.10–1.37	380–979	5.0–24	55–4361		75–421	0.136–23.54	Bergamin et al. (2009)
Bagnoli-Coroglio	Surface sediment	8000–100,000	0.5–4		0.01–3.24		4.0–54	0.5–126	2000–597,000	0.01–9.27	457–5947	0.01–53	52–896		91–2313	0.004–2.89	Romano et al. (2004)
Bagnoli-Coroglio	Surface sediment				0.1–4.8	3.0–13		9.0–95		0.03–1		3.0–25	83–775		160–1600		Sharp and Nardi 1987
Gulf of Pozzuoli	Surface sediment						15–53	19–58					48–221				Griggs and Johons (1978)

**Fig. 5:**
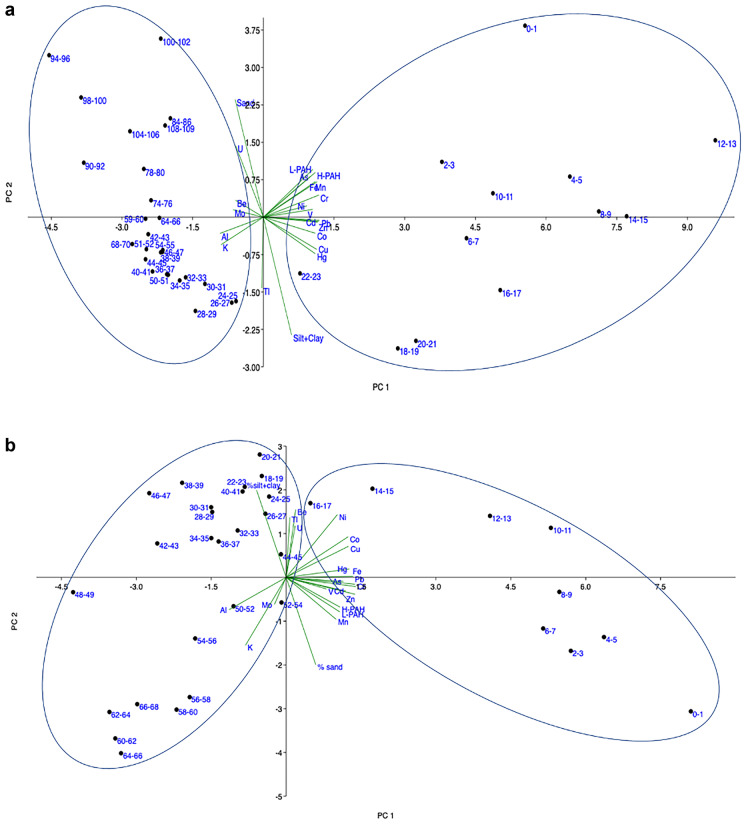
**a** PCA plot of the layers from the AB01 sediment core. The two components (PC1 and PC2) explain the 76% of the total dataset variance. The numbers used as point labels represent the layers’ depth (expressed in cm). **b** PCA plot of the layers from the AB02 sediment core. The two components (PC1 and PC2) explain the 70% of the total dataset variance. The numbers used as point labels represent the layers’ depth (expressed in cm)

Spearman’s correlations calculated among the variables (Table ESM 2) show that, on the whole, metals associated with the manufacturing activities in the Bagnoli-Coroglio area (especially the steelworks) and PAHs have strong correlations with each other, thus confirming a common origin and release into the environment over the years. On the contrary, metals not strictly related to the industrial activities in the area (such as Be, Mo, Tl, U) do not show significant correlations with the other parameters. The PCA (Fig. 5a, b) shows, for both sediment cores, a clear differentiation of the samples into two groups. For each sediment core, the group on the right side of the PCA plot collects the samples with the highest concentrations of HMs and PAHs. According to the orientation of the vectors (the segments representing the variables and their contribution to the PCA), these samples have a good correlation with the elements associated with the industrial activities carried out in the brownfield area. Indeed, considering the core AB01, the one having a well-defined dating, the samples gathered in this group are the layers dated from 1916 onwards (thus, from a few years after the beginning of industrial activities). The group on the left of the PCA graph, instead, collects samples with lower amounts of metals and PAHs; these samples are the layers deposited before the industrial exploitation of the area, and their composition is therefore related only to geogenic inputs. In both the PCA graphs the sample groups show a vertical stretched elliptic shape, which is influenced by the orientation of the vectors representing the grain-size parameters (sand and silt+clay). Indeed, the sediment fine fraction (silt+clay) has a high surface area and adsorption capacity that can significantly affect the retention of pollutants.

**Fig. 6:**
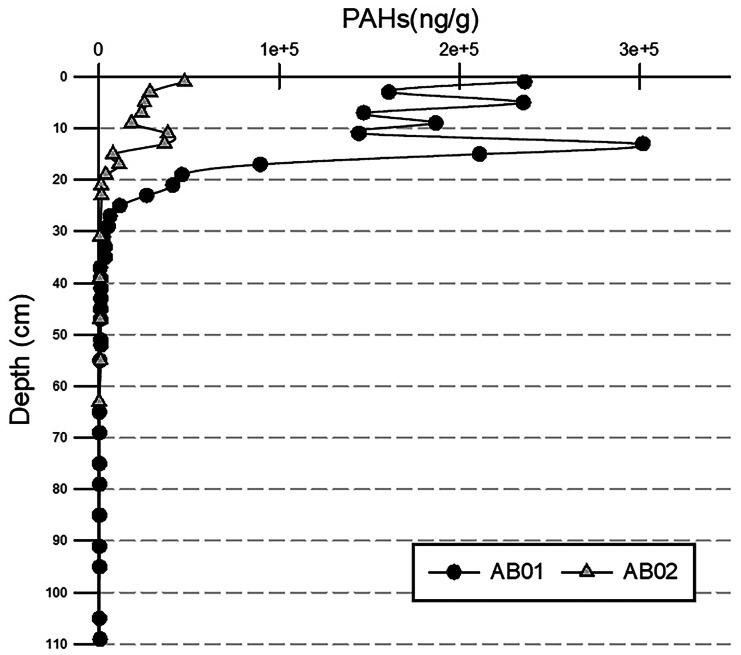
Total PAHs concentration along sediment cores AB01 and AB02

A significant difference in both total and individual PAH concentrations was observed between the two sites (higher amounts in AB01), which is related to the distance from the brownfield area (the pollution source) and the grain size composition during the industrial era (silt+clay percentages are 85 % and 73 % in core AB01 and AB02, respectively); moreover, the fine sediment moving from the industrial area toward core AB02 station, settles down at 30—40-m depth, along a wide marine terrace (Trifuoggi et al., 2017) and does not reaches the 65-m depth (AB02 station). However, despite the differences, PAH trends are similar on the time scale (Fig. 6), showing first a gradual increase from the beginning of the last century, then an exponential increase from the post-war period until the 1960s–1980s (the peak of steel production), and finally a decreasing trend to intermediate values after the end of the industrial exploitation of the area. Although dating back to periods following the end of industrial activities and the decommissioning of the site, the most superficial layers are still affected by contamination, probably due to the transport of the finest fraction from the areas of highest concentration on the site.

**Fig. 7:**
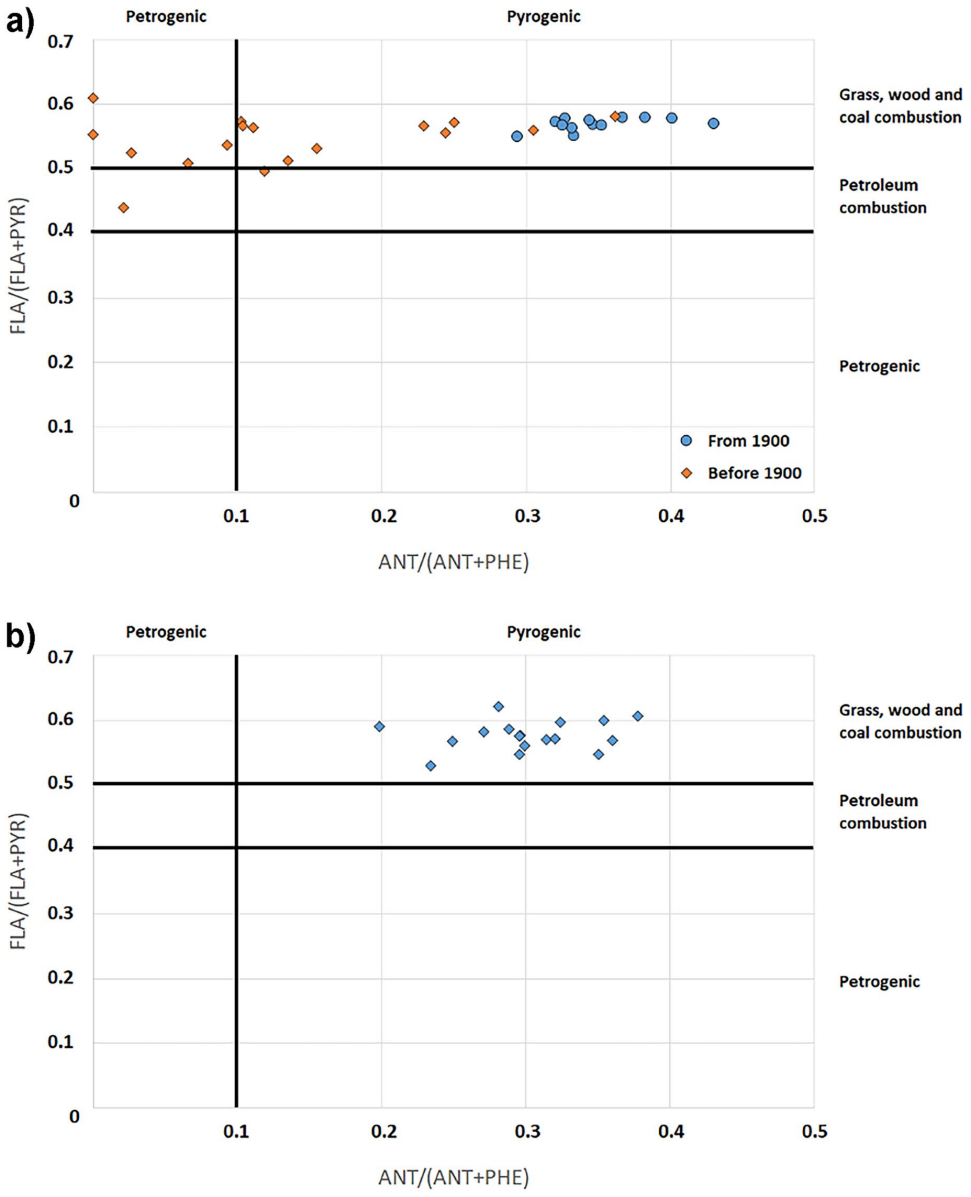
**a** PAHs diagnostic ratio of the layers from the AB01 sediment core. **b** PAHs diagnostic ratio of the layers from the AB02 sediment core

The PAHs diagnostic ratio diagrams in Fig. 7a (for AB01) and Fig. 7b (for AB02), based on the concentrations of anthracene (ANT), phenanthrene (PHE), fluoranthene (FLA), and pyrene (PYR), are useful to evaluate the origin of the organic contaminants (Tobiszewski & Namieśnik, 2012). The diagram of AB01 sediment core is particularly interesting since it shows two clusters of samples on the chart area; the group on the left side (having ANT/(ANT+PHE) ratio < 0.2) refers to sediment layers dated in the first half of 1800 (1846 and before), while the group on the right side (having ANT/(ANT+PHE) ratio > 0.2) gathers layers from the second half of 1800 (from 1856 and later). The ANT/(ANT+PHE) is associated with the combustion products, and the first industrial exploitation of the Bagnoli site, starting in 1854 with a chemical factory. The diagram also evidences the consequence of the increasing industrial activity volume, since the layers of the 1900s (blue circles in Fig. 7a), characterized by the construction and the starting of the steel-plant, show an increasing trend of the ANT/(ANT+PHE). The FLA/(FLA+PYR) ratio evidenced that the PAHs were mainly originated from the combustion of wood and coal (considering that the area was a very active industrial site, the combustion of coal was the prominent one). The diagram concerning the AB02 sediment core is less interesting, because due to the abovementioned absence of a time marker only the layers in the first 21 cm (corresponding to the range 1907–2015) were considered, and also in this case the PAHs seem to be related to combustion.

**Fig. 8:**
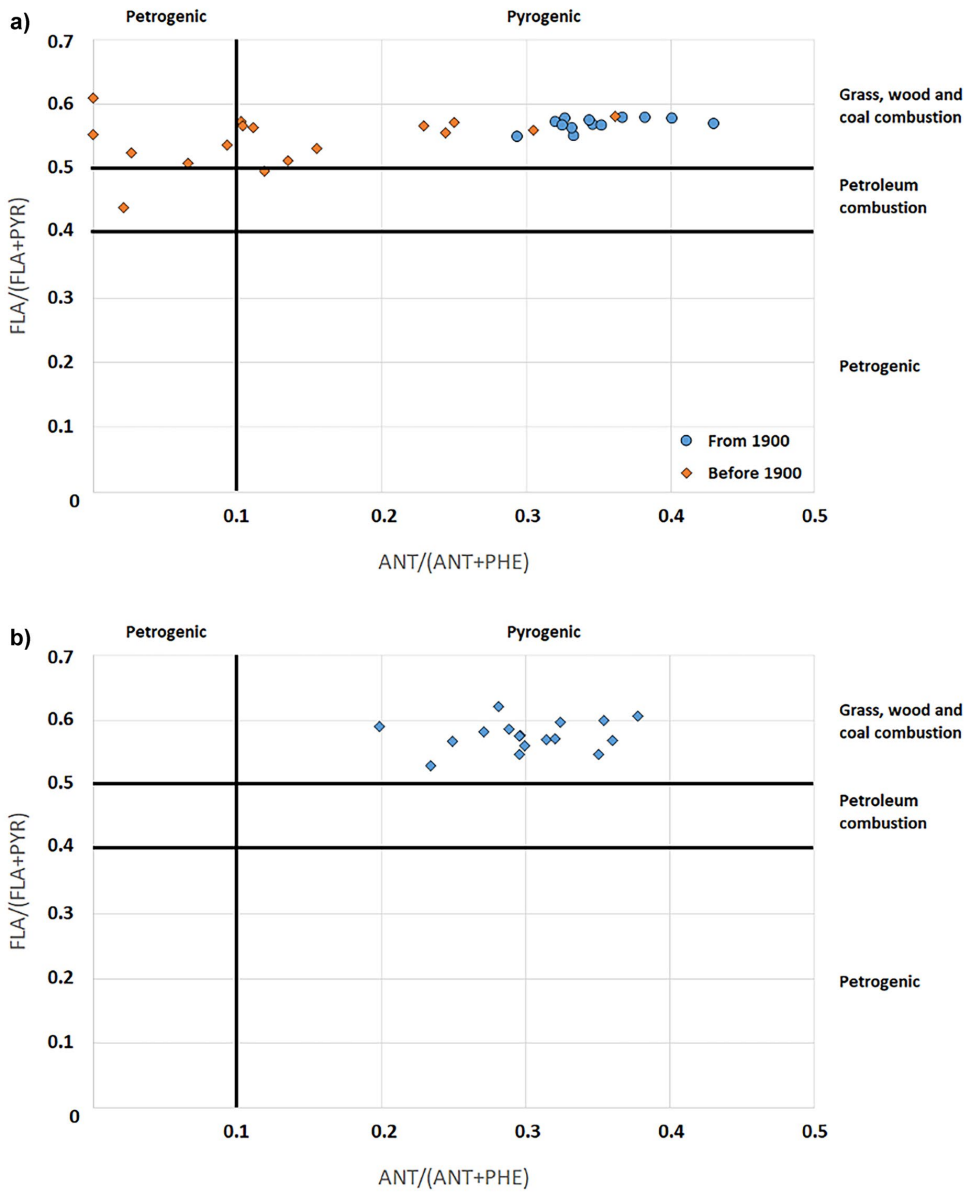
Contamination factor (Cf) profiles for AB01 and AB02 sediment cores

The Cf profiles of sediment cores AB01 and AB02 are shown in Fig. 8. On the whole, the Cf values concerning AB01 show a decreasing trend from top to bottom. Most of the elements (Al, As, Be, Co, Cr, Fe, K, Mn, Mo, Ni, Tl, U, and V) are characterized by low (Cf < 1) or moderate (1 ≤ Cf < 3) contamination levels. However, the trend for As, Be, K, Tl, and U shows a peak in enrichment from 75-cm depth, which becomes much more evident at 90-–95-cm depth. According to geochronological dating, the formation of Monte Nuovo (1538 A.D.) dates to this depth. This eruption, which represents the last event in the Phlegraean Fields (Di Vito et al., 1987), was probably the cause of the increased emission of these elements. The profile of As can be explained by the presence of hydrothermal fluids related to the volcanic activity of the Phlegraean Fields and typically present in the coastal marine area of Bagnoli (Di Vito et al., 1987). Finally, Zn, Pb, Cd, Hg, and Cu show “very high” (Cf ≥ 6) enrichment values in the surface layers (0–20 cm) and “low” enrichment values (Cf < 1) in the deeper layers of the sediment core, indicating a significant anthropogenic contribution to the onset of industrial activities at the Bagnoli site. Sediment core AB02 shows a flatter pattern and a prevalence of low levels of contamination (Cf < 1).

The overall contamination assessment was accomplished by calculating the PLI, based on the elements showing the highest enrichments (Cd, Cu, Hg, Pb, and Zn).

**Fig. 9:**
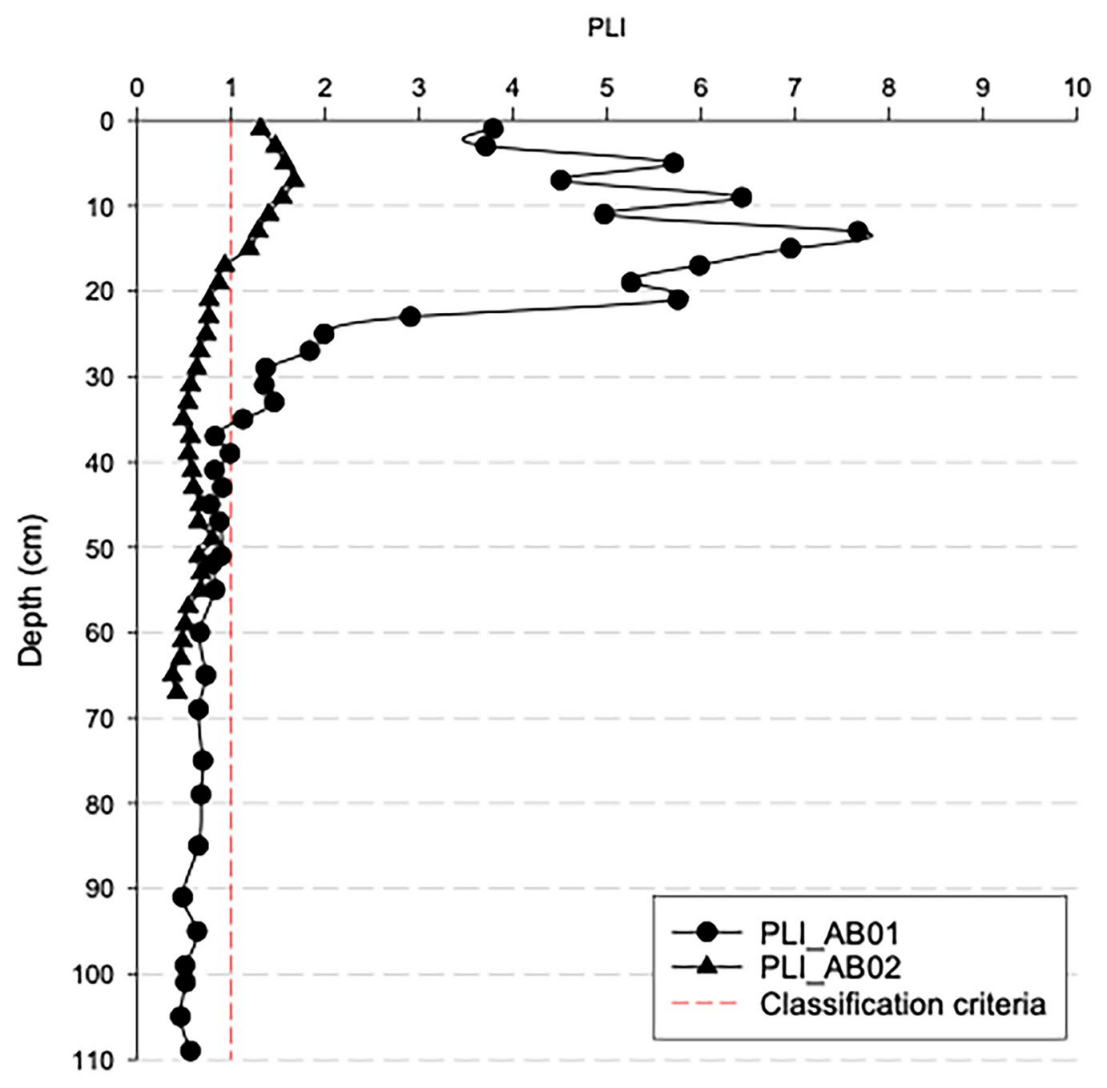
Pollution load index (PLI) profiles (calculated as overall contamination by Cd, Cu, Hg, Pb, and Zn) for AB01 and AB02 sediment cores

The PLI trends obtained for both AB01 and AB02 are shown in Fig. 9. The results confirm the previous observations made for both sediment cores. Indeed, referring to AB01 (the sediment core with a reliable dating) the most polluted layers (PLI > 1) are located in the 0–35 cm, and extremely high PLI values (> 4) were recorded in the 0–20 cm (dated from 1925 onwards and corresponding to the years of the greatest industrial exploitation of the area). The AB02 sediment core showed significantly lower PLI results, reaching values > 1 only in the 0—15-cm layers, as a consequence of its grain size composition (Fig. 4), being the AB02 richer in sand than AB01. Hence, the coastal area facing the Bagnoli-Coroglio brownfield site is strongly contaminated by metals whose distribution seems to be controlled by the grain size of the sea sediments (Albanese et al., 2010). The PLI is assumed to assess the degree to which the HMs associated with a sediment can impact flora and fauna (Tomlinson et al., 1980), so we can assume that the input of pollutants over the last century into Bagnoli Bay has potentially severely affected organisms living in contact with the sediments.

The depth profile of PLI and the total PAHs show a very analogous trend, thus further confirming a common pollution source and a simultaneous release of the pollutants during the years.

## Conclusion

Geochronological and geochemical data from two sediment cores collected from the Bagnoli brownfield site allowed for the determination of heavy metal local natural background levels (NBLs) and the investigation of heavy metals (HMs) and polycyclic aromatic hydrocarbons (PAHs) contamination temporal trends.

For the calculation of NBLs an integrated approach, based on geochemistry and statistic, allowed the discrimination between the pre-industrial (geogenic) occurrence and industrial (anthropogenic) enrichment of HMs.

The sediment core AB01 showed higher concentrations of both the two types of pollutants, mainly because of the location (closer to the brownfield site) and a grain size composition richer in fine fraction (silt+clay).

Of the studied elements, As, Be, Tl, and V, have NBL values above local regulatory limits. The origin of these elements is in fact linked to the hydrothermal activity that strongly influences the area, thus highlighting that the geogenic contribution must be taken into account for a proper assessment of anthropogenic impact.

As reported in previous studies, the concentrations of Cd, Cu, Hg, Pb, and Zn in the layers belonging to the industrial period showed high enrichments. This is also confirmed by the calculation of the contamination factor (Cf) and the pollution load index (PLI). Lower enrichments were measured for Cr, Fe, Mn, Ni, and Co.

PAHs also showed increasing concentrations since the beginning of the industrial exploitation of the Bagnoli area (early twentieth century). An exponential increase was evidenced from the post-war period until the 1960s–1980s (corresponding to the peak of steel production), followed by a decreasing trend associated with the decline of industrial activities.

Concentrations of both HMs and PAHs corresponding to the post-industrial years (surface layers) still show significant values, indicating persistent contamination reasonably associated with the fine fraction of sediments to which they were sorbed.

These data can help risk managers take decisions concerning appropriate remedial actions. This is particularly true for sediments, as their classification usually refers to generic “reference chemical levels” that represent a national average situation, not considering the case of those from areas with natural anomalies. Moreover, different sediment classes are often defined concerning their contaminant content, and each class is assigned for different purposes. Thus, without considering site-specific NBLs, sediments with natural concentrations exceeding regulatory limits would not be available for alternative uses (e.g., coastal nourishment or habitat restoration) and could become an additional disadvantage for site remediation.

## Supplementary Information

Below is the link to the electronic supplementary material.

Supplementary file1 (DOCX 15 KB)

Supplementary file2 (DOCX 31 KB)

## Data Availability

The datasets generated during and/or analyzed during the current study are available from the corresponding author on reasonable request.
