# Supplementary material for: Heavy metal background levels and pollution temporal trend assessment within the marine sediments facing a brownfield area (Gulf of Pozzuoli, Southern Italy)
Source: Environ Monit Assess. 2022 Sep 21;194(11):814. doi: 10.1007/s10661-022-10480-3 (PMC9492602; doi:10.1007/s10661-022-10480-3)
Supplement: Supplementary file 1 — Supplementary file1 (DOCX 15 KB) [file 10661_2022_10480_MOESM1_ESM.docx]

**Table ESM1** Quality check performances for the two detectors used in geochronological analysis

|  |  |  | Det 1 | | Det 2 | |
| --- | --- | --- | --- | --- | --- | --- |
| Nuclide | Photopeak | Certified value ^a^ | Measured value | MDA ^b^ | Measured value | MDA ^b^ |
|  | keV | Bq kg^-1^ | Bq kg^-1^ | Bq kg^-1^ | Bq kg^-1^ | Bq kg^-1^ |
| ^210^Pb | 46.5 | 26.0-29.2 | 27.2+-2.3 (n=4) | 4.9 | 28.1+-3.1 (n=9) | 4.8 |
| ^226^Ra | 351.9 | 21.6-22.4 | 22.6+1.2 (n=4) | 0.8 | 22.0+1.2 (n=9) | 1 |
| ^a^ Confidence Interval 95%, ^210^Pb decay corrected to measurement data  ^b^ Minimum Detectable Activity defined by Currie method | | | | | | |
