# Supplementary material for: Heavy metal background levels and pollution temporal trend assessment within the marine sediments facing a brownfield area (Gulf of Pozzuoli, Southern Italy)
Source: Environ Monit Assess. 2022 Sep 21;194(11):814. doi: 10.1007/s10661-022-10480-3 (PMC9492602; doi:10.1007/s10661-022-10480-3)
Supplement: Supplementary file 2 — Supplementary file2 (DOCX 31 KB) [file 10661_2022_10480_MOESM2_ESM.docx]

**Table ESM2** Spearman correlations for sediment core AB01 and AB02

| **Core AB01** | Be | V | Cr | Mn | Fe | Co | Ni | Cu | Zn | As | Mo | Cd | Tl | Pb | U | Hg | Al | K | Sand | Silt + Clay | L-PAHs | H-PAHs |
| --- | --- | --- | --- | --- | --- | --- | --- | --- | --- | --- | --- | --- | --- | --- | --- | --- | --- | --- | --- | --- | --- | --- |
| Be | 1 |  |  |  |  |  |  |  |  |  |  |  |  |  |  |  |  |  |  |  |  |  |
| V | -0.3 | 1 |  |  |  |  |  |  |  |  |  |  |  |  |  |  |  |  |  |  |  |  |
| Cr | -0.5 | **0.9** | 1 |  |  |  |  |  |  |  |  |  |  |  |  |  |  |  |  |  |  |  |
| Mn | -0.1 | **0.8** | **0.7** | 1 |  |  |  |  |  |  |  |  |  |  |  |  |  |  |  |  |  |  |
| Fe | -0.3 | **0.9** | **0.9** | **0.9** | 1 |  |  |  |  |  |  |  |  |  |  |  |  |  |  |  |  |  |
| Co | -0.3 | **0.9** | **0.9** | **0.8** | **0.9** | 1 |  |  |  |  |  |  |  |  |  |  |  |  |  |  |  |  |
| Ni | -0.3 | **0.6** | **0.8** | **0.6** | **0.7** | **0.7** | 1 |  |  |  |  |  |  |  |  |  |  |  |  |  |  |  |
| Cu | -0.4 | **0.7** | **0.8** | **0.7** | **0.7** | **0.8** | **0.7** | 1 |  |  |  |  |  |  |  |  |  |  |  |  |  |  |
| Zn | -0.3 | **0.7** | **0.8** | **0.8** | **0.8** | **0.8** | **0.6** | **0.9** | 1 |  |  |  |  |  |  |  |  |  |  |  |  |  |
| As | -0.3 | **0.6** | **0.6** | **0.6** | **0.6** | 0.5 | **0.7** | 0.4 | 0.5 | 1 |  |  |  |  |  |  |  |  |  |  |  |  |
| Mo | 0.4 | -0.5 | *-0.6* | -0.5 | -0.5 | *-0.6* | -0.3 | *-0.6* | *-0.6* | -0.1 | 1 |  |  |  |  |  |  |  |  |  |  |  |
| Cd | -0.2 | **0.7** | **0.7** | **0.8** | **0.8** | **0.8** | **0.6** | **0.8** | **0.9** | 0.5 | *-0.6* | 1 |  |  |  |  |  |  |  |  |  |  |
| Tl | 0.5 | 0 | 0 | 0.2 | 0.1 | 0.1 | 0 | 0.1 | 0.2 | 0 | 0.2 | 0.2 | 1 |  |  |  |  |  |  |  |  |  |
| Pb | -0.3 | **0.7** | **0.8** | **0.7** | **0.8** | **0.8** | **0.6** | **1** | **1** | 0.4 | *-0.6* | **0.9** | 0.2 | 1 |  |  |  |  |  |  |  |  |
| U | **0.6** | -0.1 | -0.3 | -0.1 | -0.2 | -0.3 | -0.3 | -0.5 | -0.4 | -0.3 | 0.4 | -0.4 | 0.2 | -0.5 | 1 |  |  |  |  |  |  |  |
| Hg | -0.4 | **0.6** | **0.7** | **0.7** | **0.7** | **0.8** | **0.6** | **1** | **0.9** | 0.4 | *-0.6* | **0.9** | 0.2 | **1** | *-0.6* | 1 |  |  |  |  |  |  |
| Al | 0.3 | *-0.6* | *-0.7* | *-0.6* | *-0.7* | *-0.6* | *-0.6* | *-0.6* | *-0.7* | *-0.6* | **0.6** | *-0.7* | 0.2 | *-0.6* | 0.4 | *-0.6* | 1 |  |  |  |  |  |
| K | 0.5 | -0.5 | *-0.7* | -0.5 | *-0.6* | *-0.6* | *-0.6* | -0.5 | -0.5 | *-0.6* | 0.5 | *-0.6* | 0.3 | -0.5 | **0.6** | -0.5 | **0.8** | 1 |  |  |  |  |
| Sand | 0.2 | -0.4 | -0.5 | -0.4 | -0.4 | *-0.6* | -0.3 | *-0.8* | *-0.8* | -0.1 | 0.5 | *-0.7* | -0.3 | *-0.8* | 0.5 | *-0.8* | 0.4 | 0.3 | 1 |  |  |  |
| Silt + Clay | -0.2 | 0.4 | 0.5 | 0.4 | 0.4 | **0.6** | 0.4 | **0.8** | **0.8** | 0.1 | -0.5 | **0.7** | 0.3 | **0.8** | -0.4 | **0.8** | -0.4 | -0.3 | *-1* | 1 |  |  |
| L-PAHs | -0.5 | **0.7** | **0.8** | **0.8** | **0.8** | **0.8** | **0.6** | **0.9** | **0.9** | 0.5 | *-0.7* | **0.8** | -0.1 | **0.9** | -0.4 | **0.9** | *-0.7* | *-0.6* | *-0.6* | **0.6** | 1 |  |
| H-PAHs | -0.5 | **0.7** | **0.8** | **0.8** | **0.8** | **0.8** | **0.6** | **0.9** | **0.9** | 0.4 | *-0.8* | **0.9** | 0 | **1** | -0.5 | **0.9** | *-0.6* | *-0.6* | *-0.7* | **0.7** | **0.9** | 1 |
| Correlation is significant at 0.01 level; correlations greater than 0.6 and lower than -0.6 are respectively typed in **bold** and *italics*. | | | | | | | | | | | | | | | | | | | | | | |

| **Core AB02** | Be | V | Cr | Mn | Fe | Co | Ni | Cu | Zn | As | Mo | Cd | Tl | Pb | U | Hg | Al | K | Sand | Silt + Clay | L-PAHs | H-PAHs |
| --- | --- | --- | --- | --- | --- | --- | --- | --- | --- | --- | --- | --- | --- | --- | --- | --- | --- | --- | --- | --- | --- | --- |
| Be | 1 |  |  |  |  |  |  |  |  |  |  |  |  |  |  |  |  |  |  |  |  |  |
| V | -0.4 | 1 |  |  |  |  |  |  |  |  |  |  |  |  |  |  |  |  |  |  |  |  |
| Cr | 0.1 | **0.6** | 1 |  |  |  |  |  |  |  |  |  |  |  |  |  |  |  |  |  |  |  |
| Mn | -0.2 | 0.4 | 0.5 | 1 |  |  |  |  |  |  |  |  |  |  |  |  |  |  |  |  |  |  |
| Fe | 0.2 | **0.6** | **0.7** | **0.6** | 1 |  |  |  |  |  |  |  |  |  |  |  |  |  |  |  |  |  |
| Co | 0.3 | 0.5 | **0.8** | 0.4 | **0.8** | 1 |  |  |  |  |  |  |  |  |  |  |  |  |  |  |  |  |
| Ni | 0.5 | 0.3 | **0.8** | 0.2 | **0.6** | **0.9** | 1 |  |  |  |  |  |  |  |  |  |  |  |  |  |  |  |
| Cu | 0.3 | 0.3 | **0.7** | 0.3 | **0.6** | **0.8** | **0.7** | 1 |  |  |  |  |  |  |  |  |  |  |  |  |  |  |
| Zn | 0.3 | 0.4 | **0.8** | 0.4 | **0.7** | **0.8** | **0.7** | **0.8** | 1 |  |  |  |  |  |  |  |  |  |  |  |  |  |
| As | 0.2 | 0.3 | 0.5 | 0.3 | **0.6** | **0.7** | **0.6** | 0.5 | **0.6** | 1 |  |  |  |  |  |  |  |  |  |  |  |  |
| Mo | -0.5 | 0.2 | 0.1 | 0.2 | -0.1 | -0.1 | -0.3 | 0.2 | 0.2 | 0 | 1 |  |  |  |  |  |  |  |  |  |  |  |
| Cd | 0 | 0.3 | 0.5 | 0.5 | 0.5 | 0.5 | 0.4 | **0.6** | **0.7** | 0.4 | 0.3 | 1 |  |  |  |  |  |  |  |  |  |  |
| Tl | 0.5 | -0.2 | 0.2 | -0.2 | 0.2 | 0.3 | 0.5 | 0.5 | 0.4 | 0.1 | -0.1 | 0.3 | 1 |  |  |  |  |  |  |  |  |  |
| Pb | 0.2 | 0.4 | **0.8** | 0.3 | **0.6** | **0.7** | **0.7** | **0.9** | **0.9** | 0.5 | 0.3 | **0.6** | 0.4 | 1 |  |  |  |  |  |  |  |  |
| U | 0.4 | 0.1 | 0.1 | 0.2 | 0.4 | 0.3 | 0.3 | 0.2 | 0.1 | 0.2 | -0.3 | 0 | 0.4 | 0.1 | 1 |  |  |  |  |  |  |  |
| Hg | 0.4 | 0.2 | **0.8** | 0.3 | **0.6** | **0.7** | **0.7** | **0.9** | **0.9** | 0.5 | 0.1 | **0.7** | 0.5 | **0.9** | 0.1 | 1 |  |  |  |  |  |  |
| Al | -0.4 | -0.3 | *-0.6* | -0.3 | *-0.7* | *-0.7* | *-0.7* | *-0.6* | *-0.6* | *-0.7* | 0.2 | -0.2 | -0.2 | -0.5 | -0.3 | -0.5 | 1 |  |  |  |  |  |
| K | -0.2 | -0.3 | -0.4 | -0.1 | -0.4 | *-0.7* | *-0.6* | *-0.6* | -0.4 | -0.4 | 0.3 | -0.2 | -0.2 | -0.5 | -0.1 | -0.5 | **0.6** | 1 |  |  |  |  |
| Sand | -0.4 | 0.3 | 0.3 | **0.6** | 0.3 | 0 | -0.1 | 0.1 | 0.3 | 0.2 | 0.4 | 0.4 | -0.4 | 0.2 | -0.2 | 0.1 | 0 | 0.3 | 1 |  |  |  |
| Silt + Clay | 0.4 | -0.3 | -0.3 | *-0.6* | -0.3 | 0 | 0.1 | -0.1 | -0.3 | -0.2 | -0.4 | -0.4 | 0.4 | -0.2 | 0.2 | -0.1 | 0 | -0.3 | *-1* | 1 |  |  |
| L-PAHs | 0 | **0.6** | **0.9** | **0.7** | **0.9** | **0.9** | **0.7** | **0.8** | **0.9** | **0.6** | 0.2 | **0.7** | 0.2 | **0.9** | -0.1 | **0.9** | *-0.8* | *-0.7* | **0.6** | *-0.6* | 1 |  |
| H-PAHs | 0 | **0.6** | **0.9** | **0.7** | **0.9** | **0.9** | **0.7** | **0.8** | **0.9** | **0.6** | 0.1 | **0.7** | 0.2 | **0.9** | -0.1 | **0.9** | *-0.8* | *-0.7* | **0.6** | *-0.6* | 1 | 1 |
| Correlation is significant at 0.01 level; correlations greater than 0.6 and lower than -0.6 are respectively typed in **bold** and *italics*. | | | | | | | | | | | | | | | | | | | | | | |
